# Supplementary material for: Long-term intensive golf training induces reconfiguration of brain structural covariance networks
Source: PLoS One. 2026 Mar 9;21(3):e0344165. doi: 10.1371/journal.pone.0344165 (PMC12970903; doi:10.1371/journal.pone.0344165)
Supplement: Attachment — Submitted filename: PONE-D-25-36556.pdf [file pone.0344165.s001.pdf]

PLOS ONE

Long-Term Intensive Golf Training Induces Reconfiguration of brain structural covariance networks

--Manuscript Draft--

**Manuscript Number:**	PONE-D-25-36556
**Article Type:**	Research Article
**Full Title:**	Long-Term Intensive Golf Training Induces Reconfiguration of brain structural covariance networks
**Short Title:**	Brain Network Reorganization in Expert Golfers
**Corresponding Author:**	Qiangqin Song<br>Beijing Normal University<br>Beijing, CHINA
**Keywords:**	golfer；brain structure； structural covariance network
**Abstract:**	This study explores how long-term intensive golf training influences the reorganization of brain structural covariance networks (SCNs) by employing an individualized network-based approach. A total of 60 participants—30 expert golfers, 30 novice golfers, and 30 non-golfer controls—underwent T1-weighted MRI scans to construct individual SCNs using inter-regional effect size differences (ESD). Network metrics such as clustering coefficient (Cp), local efficiency (Eloc), characteristic path length (Lp), and global efficiency (Eg) were calculated across multiple sparsity thresholds to evaluate topological differences. At the global level, expert golfers exhibited significantly higher Cp and Eloc compared to novices, suggesting enhanced modularity and localized information processing. Nodal analysis revealed increased clustering and local efficiency in brain regions associated with visual and sensorimotor integration, including the right supramarginal gyrus, Heschl's gyrus, and left middle temporal pole. In contrast, experts showed reduced global efficiency in the left calcarine cortex and altered path length in the right cerebellum-10. Furthermore, mediation analyses demonstrated that the clustering coefficient mediated the relationship between training duration and golf performance, particularly stroke accuracy. These findings indicate that prolonged motor training leads to adaptive brain network reconfigurations that favor modular specialization and efficient neural processing, supporting superior sensorimotor skills in expert athletes. This study provides novel insights into the neuroplastic mechanisms underlying motor expertise and highlights the importance of integrating both global and regional network analyses in understanding skill acquisition.
**Order of Authors:**	Zong han Lei
	Yao qi Hou
	Qiangqin Song
**Additional Information:**	
**Question**	**Response**
**Financial Disclosure**<br><br>Enter a financial disclosure statement that describes the sources of funding for the work included in this submission. Review the submission guidelines for detailed requirements. View published research articles from *PLOS ONE* for specific examples.<br><br>This statement is required for submission	The author(s) received no specific funding for this work.

and **will appear in the published article** if the submission is accepted. Please make sure it is accurate.<br><br>**Funded studies**<br>Enter a statement with the following details:<br>• Initials of the authors who received each award<br>• Grant numbers awarded to each author<br>• The full name of each funder<br>• URL of each funder website<br>• Did the sponsors or funders play any role in the study design, data collection and analysis, decision to publish, or preparation of the manuscript?<br><br>Did you receive funding for this work?	
**Competing Interests**<br><br>Use the instructions below to enter a competing interest statement for this submission. On behalf of all authors, disclose any competing interests that could be perceived to bias this work—acknowledging all financial support and any other relevant financial or non-financial competing interests.<br><br>This statement is required for submission and **will appear in the published article** if the submission is accepted. Please make sure it is accurate and that any funding sources listed in your Funding Information later in the submission form are also declared in your Financial Disclosure statement.<br><br>View published research articles from *PLOS ONE* for specific examples.	The authors have declared that no competing interests exist. We confirm that there are no financial, personal, or professional interests that could be perceived to influence the objectivity of this research. All sources of funding supporting the work described in this manuscript have been disclosed in the Funding Information section.

**NO authors have competing interests**<br><br>Enter: *The authors have declared that no competing interests exist.*<br><br>**Authors with competing interests**<br><br>Enter competing interest details beginning with this statement:<br><br>*I have read the journal's policy and the authors of this manuscript have the following competing interests: [insert competing interests here]*<br><br>* typeset	
**Ethics Statement**<br><br>Enter an ethics statement for this submission. This statement is required if the study involved:<br><br>• Human participants<br>• Human specimens or tissue<br>• Vertebrate animals or cephalopods<br>• Vertebrate embryos or tissues<br>• Field research<br><br>Write "N/A" if the submission does not require an ethics statement.<br><br>General guidance is provided below. Consult the submission guidelines for detailed instructions. **Make sure that all information entered here is included in the Methods section of the manuscript.**	All participants were right-handed, had normal or corrected-to-normal vision, no history of neurological or psychiatric disorders, and met the safety requirements for MRI scanning. Participant recruitment was conducted from 01/05/2023 to 15/12/2023. This study was approved by the Ethics Committee of the College of Physical Education and Sport Sciences, Beijing Normal University (Project Number: 133325607). Written informed consent was obtained from all participants. The research materials and findings are strictly used for scientific research purposes and do not involve any conflicts of interest. The experimental design and protocol were reviewed and deemed to adequately consider principles of safety and fairness by the Ethics Committee. Participant recruitment was based on voluntary participation, and their rights and privacy were fully protected.

**Format for specific study types**<br><br>**Human Subject Research (involving human participants and/or tissue)**<br>• Give the name of the institutional review board or ethics committee that approved the study<br>• Include the approval number and/or a statement indicating approval of this research<br>• Indicate the form of consent obtained (written/oral) or the reason that consent was not obtained (e.g. the data were analyzed anonymously)<br><br>**Animal Research (involving vertebrate animals, embryos or tissues)**<br>• Provide the name of the Institutional Animal Care and Use Committee (IACUC) or other relevant ethics board that reviewed the study protocol, and indicate whether they approved this research or granted a formal waiver of ethical approval<br>• Include an approval number if one was obtained<br>• If the study involved *non-human primates*, add *additional details* about animal welfare and steps taken to ameliorate suffering<br>• If anesthesia, euthanasia, or any kind of animal sacrifice is part of the study, include briefly which substances and/or methods were applied<br><br>**Field Research**<br><br>Include the following details if this study involves the collection of plant, animal, or other materials from a natural setting:<br>• Field permit number<br>• Name of the institution or relevant body that granted permission	
**Data Availability**<br><br>Authors are required to make all data underlying the findings described fully available, without restriction, and from the time of publication. PLOS allows rare exceptions to address legal and ethical concerns. See the PLOS Data Policy and FAQ for detailed information.	No - some restrictions will apply

A Data Availability Statement describing where the data can be found is required at submission. Your answers to this question constitute the Data Availability Statement and **will be published in the article**, if accepted.<br><br>**Important:** Stating 'data available on request from the author' is not sufficient. If your data are only available upon request, select 'No' for the first question and explain your exceptional situation in the text box.<br><br>Do the authors confirm that all data underlying the findings described in their manuscript are fully available without restriction?	
**Describe where the data may be found in full sentences. If you are copying our sample text, replace any instances of XXX with the appropriate details.**<br><br>• If the data are **held or will be held in a public repository**, include URLs, accession numbers or DOIs. If this information will only be available after acceptance, indicate this by ticking the box below. For example: *All XXX files are available from the XXX database (accession number(s) XXX, XXX.).*<br>• If the data are all contained **within the manuscript and/or Supporting Information files**, enter the following: *All relevant data are within the manuscript and its Supporting Information files.*<br>• If neither of these applies but you are able to provide **details of access elsewhere**, with or without limitations, please do so. For example:<br><br>*Data cannot be shared publicly because of [XXX]. Data are available from the XXX Institutional Data Access / Ethics Committee (contact via XXX) for researchers who meet the criteria for access to confidential data.*<br><br>*The data underlying the results presented in the study are available from (include the name of the third party*	Due to ethical restrictions related to participant confidentiality and data protection regulations, the full dataset cannot be made publicly available. However, de-identified data can be made available upon reasonable request to the corresponding author, subject to approval by the relevant institutional review board

and contact information or URL).<br>• This text is appropriate if the data are owned by a third party and authors do not have permission to share the data.<br><br>* typeset	
Additional data availability information:	

Powered by Editorial Manager® and ProduXion Manager® from Aries Systems Corporation

Manuscript Click here to access/download;Manuscript;Manuscript.docx

Long-Term Intensive Golf Training Induces Reconfiguration of brain structural covariance networks

Zonghan Lei[1], Yaoqi Hou, Qiangqin Song[2]

1 College of P.E. and Sports, Beijing Normal University, Beijing 100875, China

2 business school, University of New South Wales, UNSW Sydney High St Kensington NSW 2052, Australia

*Corresponding Author

E-mail: u252016415@163.com

Abstract

This study explores how long-term intensive golf training influences the reorganization of brain structural covariance networks (SCNs) by employing an individualized network-based approach. A total of 60 participants—30 expert golfers, 30 novice golfers, and 30 non-golfer controls—underwent T1-weighted MRI scans to construct individual SCNs using inter-regional effect size differences (ESD). Network metrics such as clustering coefficient (Cp), local efficiency (Eloc), characteristic path length (Lp), and global efficiency (Eg) were calculated across multiple sparsity thresholds to evaluate topological differences. At the global level, expert golfers exhibited significantly higher Cp and Eloc compared to novices, suggesting enhanced modularity and localized information processing. Nodal analysis revealed increased clustering and local efficiency in brain regions associated with visual and sensorimotor integration, including the right supramarginal gyrus, Heschl's gyrus, and left middle temporal pole. In contrast, experts showed reduced global efficiency in the left calcarine cortex and altered path length in the right cerebellum-10. Furthermore, mediation analyses demonstrated that the clustering coefficient mediated the relationship between training duration and golf performance, particularly stroke accuracy. These findings indicate that prolonged motor training leads to adaptive brain network reconfigurations that favor modular specialization and efficient neural processing, supporting superior sensorimotor skills in expert athletes. This study provides novel insights into the neuroplastic mechanisms underlying motor expertise and highlights the importance of integrating both global and regional network analyses in

understanding skill acquisition.

Keywords

golfer, brain structure, structural covariance network

1. Introduction

Motor skill learning, characterized by gradual improvements in movement speed and accuracy through repeated practice, is a critical component of athletic training[1]. This learning process typically involves an initial phase of rapid improvements followed by a prolonged period of slower, incremental gains[2]. The impact of motor skill training on brain plasticity, particularly structural changes in gray matter, has been a major focus in neuroscience and sports science[3]. Athletes undergoing intensive training from a young age often develop superior motor skills, including enhanced coordination, agility, strength, and endurance[4]. These skills are supported by long-term changes in brain structure, particularly in areas related to sensory-motor integration and cognitive control.

Early research on brain plasticity in response to motor training often focused on isolated brain regions, revealing plasticity of gray matter volume in specific areas associated with skill-related functions[5-7]. The change directions of gray matter volume are not consistent. For example, studies on London taxi drivers found significant enlargement of the hippocampus, linked to their extensive navigational experience[8]. Additionally, research has shown that athletes such as basketball players and soccer players exhibit larger gray matter volumes in the cerebellum and motor-related cortices compared to novices, suggesting that long-term training induces structural brain changes that support expert performance[9-11]. However, some research showed that in professional ballet dancers, a decrease in gray matter volume has been observed in the sensorimotor cortex, including regions involved in motor control and proprioception, such as the premotor cortex and supplementary motor area[12, 13]. This reduction might be indicative of refined and highly efficient motor processing in these regions. Additionally, in elite gymnasts, a similar reduction in gray matter has been noted in the visual cortex, particularly in regions associated with spatial awareness and coordination, such as the occipital lobe[14, 15]. The decrease in gray matter volume in these areas could reflect the streamlined neural pathways that support the advanced visual-motor integration

required in gymnastics.

Although these studies provide valuable insights into the effects of training on specific brain regions, they often rely on univariate analyses such as voxel-based morphometry (VBM), which focus on morphological changes in isolated regions. This approach overlooks the complex, interconnected nature of brain function. Recent advances in network neuroscience suggest that brain regions do not operate independently but interact as part of broader structural and functional networks[16, 17]. Understanding how long-term intensive training reconfigures these networks, rather than just individual regions, offers a more comprehensive view of the neural adaptations underlying expert motor skills.

This study addresses the limitations of previous research by employing a network-based approach to examine how long-term intensive golf training affects the structural covariance networks of the brain. By constructing individualized brain structural covariance networks, this study moves beyond traditional group-level analyses, allowing for the exploration of network reconfiguration at both global and modular levels. This approach provides a deeper understanding of how expert training reshapes brain structure, offering new insights into the neural mechanisms that differentiate experts from novices. Through this innovative framework, the study aims to bridge the gap between regional brain changes and their broader network implications, enhancing our understanding of the neural basis of expert performance in sports.

2. Subject and Method

2.1. Subject

A total of 60 participants were recruited for this study and were divided into three distinct groups: the expert golfer group, the novice golfer group, and the control group. The expert group consisted of 20 highly skilled and experienced golfers, with an average score of 72.05 ± 4.3 strokes per 18 holes. These individuals trained at least six times per week, with each session lasting a minimum of two hours, and had accumulated a minimum of 10 years of training experience. The novice group also included 20 participants who had limited golf training experience, with an average score of 86.15 ± 5.63 strokes per 18 holes. They had less than three years of training experience and practiced approximately three times per week, with each session lasting about 1.5 hours. The classification of participant expertise was based on criteria adapted from previous studies [18]. The control group

comprised 20 individuals with no prior formal golf training. All groups were matched in terms of age, gender, and educational background. No statistically significant differences were observed among the three groups regarding age or body mass index (BMI) (see Table 1). All participants were right-handed, had normal or corrected-to-normal vision, no history of neurological or psychiatric disorders, and met the safety requirements for MRI scanning. Participant recruitment was conducted from 01/05/2023 to 15/12/2023. This study was approved by the Ethics Committee of the College of Physical Education and Sport Sciences, Beijing Normal University (Project Number: 133325607). Written informed consent was obtained from all participants. The research materials and findings are strictly used for scientific research purposes and do not involve any conflicts of interest. The experimental design and protocol were reviewed and deemed to adequately consider principles of safety and fairness by the Ethics Committee. Participant recruitment was based on voluntary participation, and their rights and privacy were fully protected.

Table 1 Demographic and information of subjects

Group	Pro(n=30)	New(n=30)	Control(n=30)
Age	27.4±3.1	27.1±2.8	27.6±2.2
BMI	20.5±1.6	21.3±1.8	21.9±1.2
Strokes per 18 holes	72.05±4.3	86.15±5.63	
Training duration	12.4±1.2	1.8±1.1	

2.2. Image acquisition and preprocessing

All the subjects in this study were scanned with a Siemens 3.0T MRI scanner (Trio Tim, Erlangen, Germany) using an eight-channel head coil, including high-resolution T1 weighted imaging (T1WI). Te parameters were as follows: T1WI using a Magnetization Prepared Rapid Gradient Echo (MPRAGE) sequence, TR/TE=2250/2.6 ms, slice thickness=1 mm, fip angle=9°, FOV=256×256 mm2, and matrix size=256×256.

The present study utilized high-resolution T1-weighted imaging data, which underwent preprocessing with the CAT12 toolbox of the SPM12 software package in order to eliminate motion and other artifacts. CAT12 is considered to be more accurate than VBM8 for analysis of volume changes. All participants' images were segmented into white matter, gray matter, and cerebrospinal fluid. Subsequently, the images were normalized to the Montreal Neurological Institute (MNI)

standard space, and modulated using the Difeomorphic Anatomical Registration Trough Exponential Lie Algebra toolbox. Finally, the rest gray matter images were smoothed with a Gaussian kernel of 8 mm full-width at half maximum.

2.3. Network Constructing

As the traditional group-level structural covariance network(SCN) lost the individual network information, here we adapted a recently established approach based on the inter-regional effect size difference (ESD) to obtain individual SCN for our expert-novice study[19, 20]. To retain individual network information, we used an approach based on inter-regional effect size difference (ESD) to obtain individual SCN for our case-control study. The main steps to construct individual SCN for each participant within each cohort are as follows: (1) Regional gray matter volume(GMV) for each region of interest were adjusted for covariates (e.g., age, gender,, and total intracranial volume), and the resulting regional GMV residuals were extracted. (2) A group-based SCN ($SCN_{conotrol}$) was created across the entire control group by calculating the Pearson correlation coefficient between the regional GMV residuals for each pair of brain regions. (3) We computed the mean ($M_{control}$) and standard deviation ($SD_{control}$) values of each brain region from the control group. (4) The individual weight matrix (W) was derived from the inter-regional ESD between a single subject and the average of the control group. (5) The final individual correlation coefficient matrix for a single subject was then calculated by element-wise multiplication between W and $SCN_{conotrol}$. Using this method, we obtained a 116 × 116 connection matrix for each subject in the golf expert and novice groups.

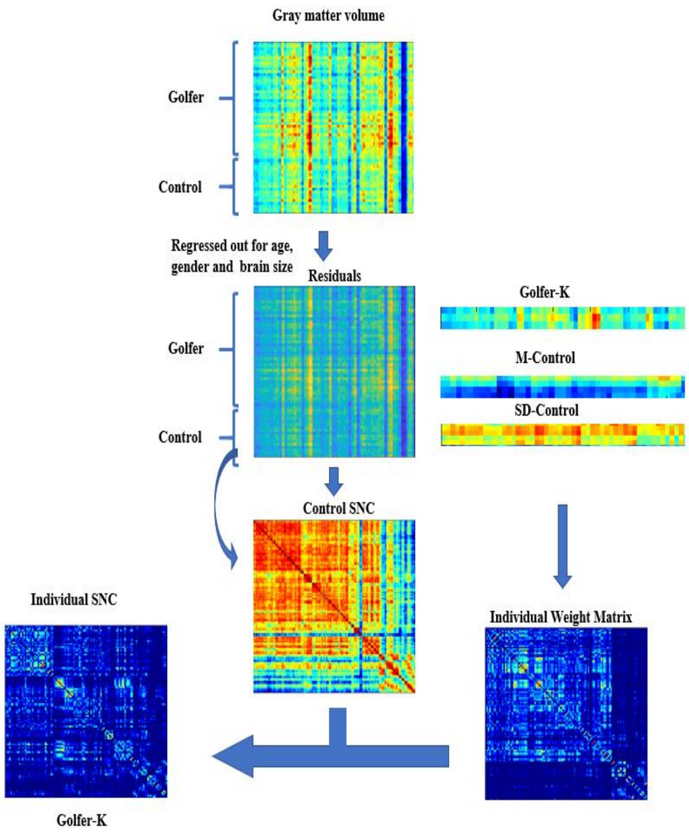

Fig 1. Schematic workflow of the contribution of structural covariance network. M, mean; SD, standard deviation; SCN, structural covariance networks.

2.4. Network analysis

A range of sparsity levels (K = 0.14–0.50, step size = 0.01) was applied to binarize individual structural covariance networks (SCNs), which were then used for group comparisons of both global and nodal network properties. The sparsity range was determined based on established criteria from previous studies (Jiang et al., 2022; Yun et al., 2020), ensuring that: (1) over 90% of nodes remained interconnected within each network, and (2) more than 95% of individual SCN graphs exhibited a small-world index greater than 1, indicating prominent small-world characteristics. At each sparsity level, four global network metrics were calculated: global efficiency (Eg), local efficiency (Eloc), average path length (Lp), and clustering coefficient (Cp). In addition, two regional (nodal) topological measures—nodal degree and betweenness centrality—were also computed for each node using the Brain Connectivity Toolbox[21]. To capture the overall performance of each topological property across the full range of sparsity thresholds, the area under the curve (AUC) was calculated for every metric and subsequently used in the statistical analyses.

Table 1 the description of global network parameters.

Network parameters	Descriptions
Clustering coefficient	It indicates the extent of local interconnectivity or cliquishness in a network.
Average path length	It measures a harmonic mean length between pairs and quantifies the ability for information propagation in parallel.
Global efficiency	It is the inverse of the harmonic mean of the characteristic path length between each pair of nodes within the network. E_{glob} measures the global efficiency of parallel information transfer in the network.
Local efficiency	It is defined as the average of the values of local efficiency across all nodes.

Mediation analysis

To further explore the interrelationships among training duration, brain structural covariance network properties, and pole performance, we conducted a mediation analysis. Drawing upon findings from Li et al., we hypothesized that neuroplasticity—reflected in changes in brain network organization—plays a mediating role in linking training duration to specific behavioral outcomes.

First, we performed correlation analyses to identify which network metrics showed significant associations with both training duration and pole performance. These network parameters were then selected as potential mediators in the subsequent mediation models. Specifically, traditional mediation involved estimating paths, including direct (path c) and indirect effects (path c') of the independent variable X on the dependent variable Y(Fig 2). The direct effect of X on Y after considering the mediator M (path c') and the indirect effect through M (i.e., the product of X → M and M → Y paths). Mediation was assessed using nonparametric bootstrapping (5000 samples) to generate bias-corrected 95% confidence intervals (CIs) for the a × b sampling distribution. A CI excluding zero indicates a significant deviation from zero for the indirect effect (path a × b, $P < .05$).

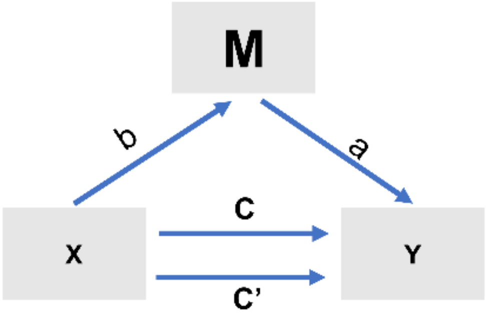

Fig 2. standard 3-variable path model of mediation analysis

2.5. Statistical analysis

We first conducted permutation tests to assess the mean differences between groups. For each network metric, we calculated the observed group difference (e.g., difference in means). Subsequently, we performed 5000 random permutations of the group labels to generate a null distribution of mean differences. The p-value for each comparison was determined as the proportion of permuted differences that were equal to or more extreme than the observed difference. This non-parametric approach provided an unbiased assessment of statistical significance while accounting for the specific data distribution. To correct for multiple comparisons across all tested metrics and brain regions, we applied the false discovery rate (FDR) correction (Benjamini & Hochberg, 1995). For correlational analysis, normal distribution……， otherwise spearman

3. Results

3.1. Alterations in global network topologies in soccer experts

The comparison of global network topologies between golf experts and novices revealed significant differences. Specifically, golf experts exhibited a higher Cp and greater Eloc in their brain structural covariance networks compared to novices(Fig 3, Table 2).

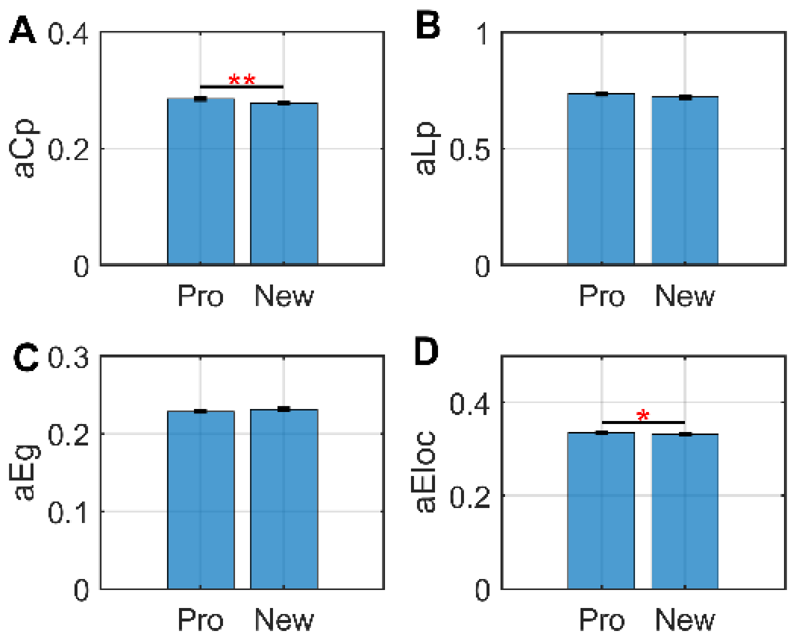

Fig 3. Comparison of global network topological properties between golf experts and novices. "*" indicates significant difference at p < 0.05, and "**" indicates p < 0.01, both after False Discovery Rate (FDR) correction. The prefix "a" in aCp , aLp , aEg , and aEloc refers to the area under the curve of the corresponding network metric.

Table 2 Comparison of global network topological properties between golf experts and novices.

	Pro	New	**P-FDR**
aCp	0.2850±0.0061	0.2786±0.0057	<0.05
aLp	0.7336±0.019	0.72±0.0221	>0.05
aEloc	0.3363±0.0046	0.3322±0.0047	<0.01
aEg	0.2282±0.0049	0.2313±0.0057	>0.05

3.2. Alteration in regional network topologies in soccer experts

At the nodal level, distinct differences were observed between experts and novices(Fig 4). Golf experts demonstrated a higher clustering coefficient in the right supramarginal gyrus, Heschl's gyrus, and the left middle temporal pole. Additionally, greater local efficiency was noted in the left middle temporal pole. Conversely, experts exhibited lower global efficiency in the left calcarine cortex and the middle temporal pole, along with a shorter path length in the right cerebellum-10 compared to novices.

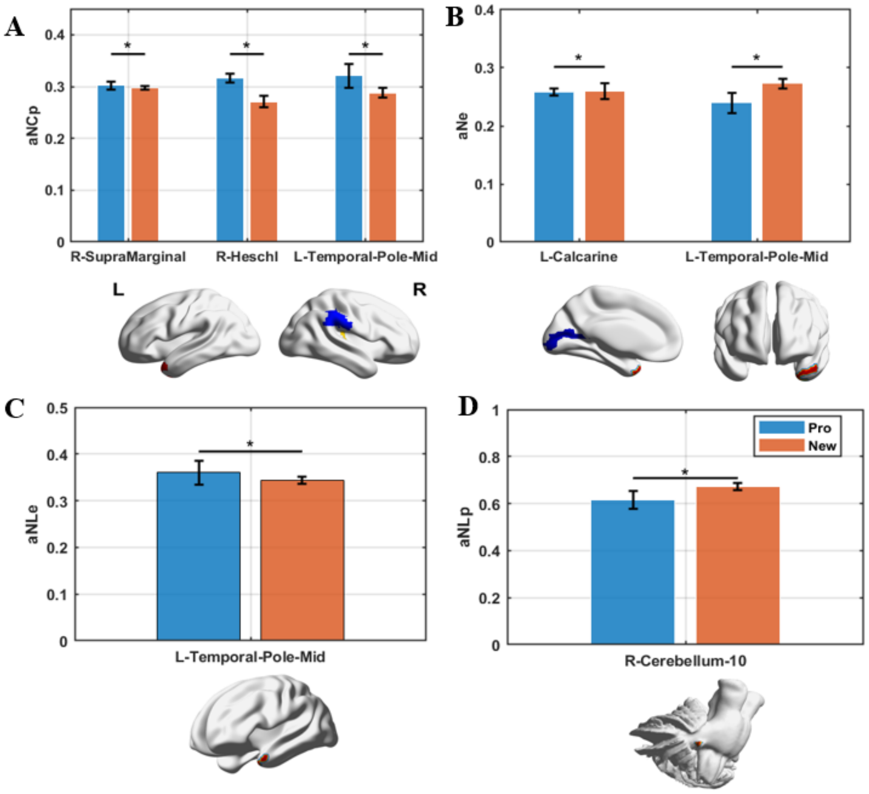

Fig 4. Group differences in nodal-level network topological properties between golf experts and novices. “*” indicates significant differences at p < 0.05 after FDR correction. The prefix “aN” in aNCp , aNLp , aNLe , and aNe denotes the area under the curve of the corresponding nodal network metric (Cp : clustering coefficient, Lp : path length, Le : local efficiency, Ne : nodal efficiency).

3.3. Mediated analysis results

Firstly, among the functional brain network parameters demonstrating significant between-group differences, we observed that global Cp and nodal Eloc of the left middle temporal pole were significantly and negatively correlated with stroke performance (Fig. 5). Furthermore, the mediated analysis indicated that training duration significantly and positively predicted aCp ($\beta = 0.33$, $p < .001$), while aCp significantly and negatively predicted the number of strokes per round ($\beta = -0.10$, $p < .05$). Meanwhile, mediation analysis revealed a significant indirect effect of aCp (point estimate = -0.22, 95% CI [-0.37, -0.09]). Moreover, when controlling for aCp, the direct effect of training duration on stroke count was no longer statistically significant ($\beta = -0.10$, $p = .15$).

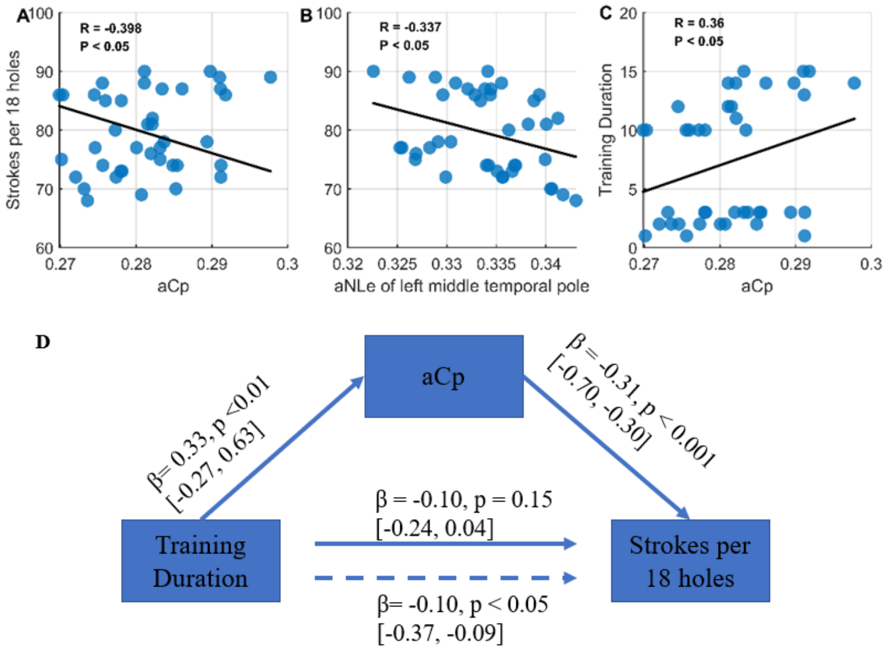

Fig 5. The relationship among structural covariance network topologies, strokes per 18 holes and training duration. Subplot A-C are the results of correlational analysis. Subplot D is the results of mediated analysis.

4. Discussion

The present study investigated structural covariance networks in expert and novice golfers, revealing distinct topological patterns associated with long-term motor expertise. At the global network level, experts exhibited enhanced organizational efficiency, as evidenced by increased clustering coefficient and local efficiency, suggesting a more specialized and modular brain architecture. At the nodal level, significant alterations were observed in key sensorimotor and cognitive-related regions—including the right supramarginal gyrus, Heschl's gyrus, left middle temporal pole, left calcarine cortex, and right cerebellum Crus 10—across multiple network metrics such as clustering coefficient, global efficiency, local efficiency, and characteristic path length. Moreover, mediation analysis revealed that the Cp significantly mediated the relationship between training duration and golf performance (i.e., average strokes per round). These findings collectively highlight the adaptive reorganization of structural brain networks associated with prolonged motor skill acquisition, underscoring both global and regional neuroplastic changes induced by extensive golf training.

At the global network level, experts exhibited enhanced organizational efficiency, as evidenced by increased clustering coefficient and local efficiency—indicative of a more specialized and

modular brain architecture. This finding aligns with previous studies suggesting that modular organization supports efficient information processing through functionally segregated subsystems[22, 23]. The observed increase in global modularity among golf experts may reflect an adaptive response to prolonged motor training, wherein the brain optimizes its functional specialization while minimizing metabolic and computational costs.

At the nodal level, significant alterations were identified in several sensorimotor and cognitive-related regions—including the right supramarginal gyrus, Heschl’s gyrus, left middle temporal pole, left calcarine cortex, and right cerebellum 10—across multiple network metrics such as clustering coefficient, global efficiency, local efficiency, and characteristic path length. These findings suggest that expertise is not only reflected in global network properties but also manifests in region-specific reorganization, potentially reflecting domain-specific skill acquisition. For instance, the supramarginal gyrus has been implicated in visuomotor integration and body schema updating[24, 25], which are essential for precise swing control and postural adjustments during golf performance.

Moreover, mediation analysis revealed that the Cp significantly mediated the relationship between training duration and golf performance (i.e., average strokes per round). This suggests that structural network reconfiguration, particularly at the local level, serves as a neural substrate underlying behavioral improvements. Importantly, this mediation effect highlights the potential utility of Cp as a neurobiological marker for tracking expertise development—a perspective that could inform future longitudinal or intervention-based studies on skill acquisition.

Importantly, our network-based approach offers a novel perspective that goes beyond traditional studies focusing on isolated brain regions. Rather than examining localized volume or activity changes, we emphasize how extensive practice reshapes interregional structural covariance across the entire brain network. This systems-level view supports the hypothesis that expertise is not merely a function of isolated neural hypertrophy but rather an emergent property of optimized, coordinated interactions among distributed brain regions[26]. Such structural adaptations likely underpin the superior motor control, sensory integration, and visuospatial processing abilities characteristic of expert performers[27].

Our findings are consistent with previous research on brain functional networks in athletes, which have reported similar modularity and efficiency patterns in individuals such as soccer players and endurance athletes—those with higher local efficiency and clustering coefficients [15, 16].

Extending these observations to structural covariance networks, our results reinforce the idea that both functional and structural architectures undergo parallel reorganization in response to intensive skill acquisition. Although structural connectivity provides the anatomical substrate for functional dynamics, the precise mechanisms linking structure to function remain incompletely understood[28-30]. Future multimodal neuroimaging studies integrating structural, functional, and behavioral data will be essential for unraveling this complex relationship.

It has been proposed that the increased modularity observed in experts' brain networks may reflect a shift toward more parallelized processing, where distinct cognitive functions—such as visuospatial attention, motor planning, and sensory integration—are supported by relatively independent neural modules[31, 32]. This organizational strategy could facilitate faster and more efficient information processing, reduce cross-talk between subsystems, and enhance overall task performance. Such an architecture is particularly advantageous in golf, a sport that demands high levels of focused attention, precise motor control, and consistent performance under varying environmental and psychological conditions.

Meanwhile, at the nodal level, the reduced global efficiency observed in certain regions (e.g., the left calcarine cortex and middle temporal pole) further suggests that expert brains may prioritize local specialization over widespread communication, aligning with the concept of "neural efficiency" previously described in cognitive neuroscience literature[33]. Similarly, consistent with our findings, prior studies have reported increased gray matter volume in these regions among athletes, supporting the notion that sustained, domain-specific training drives structural plasticity in functionally relevant cortical and subcortical areas[25, 27, 34, 35].

Notably, Yuan et al. demonstrated that golf experts exhibit significantly greater gray matter volume in the left middle temporal pole, which was positively correlated with stroke performance[18]. The temporal pole is primarily functionally connected with higher-order visual cortical regions[36]. Notably, the distinct connectivity between the temporal pole and the vestibular nuclei suggests a potential role for the temporal pole in modulating eye movement control. Furthermore, its connection to the anterior hippocampus—an area implicated in vestibular processing in humans[37]—indicates integrative involvement in sensorimotor coordination. These findings suggest that the temporal pole may regulate vestibular influence on ocular motor responses based on prior visual recognition of objects requiring interaction [28]. Additionally, the left temporal

pole has been identified as a key region for high-level sensory integration, particularly in processing semantic information[38, 39].

5. Limitations

Despite the innovative insights provided by this study, several limitations should be acknowledged. First, the cross-sectional design limits our ability to establish causality between long-term training and the observed changes in brain networks. Longitudinal studies are needed to clarify the causal relationships and track the time course of network reconfiguration in response to intensive training.

Another limitation lies in our exclusive reliance on structural MRI data. While structural covariance networks offer valuable insights into brain organization, they do not fully capture the functional dynamics that underpin expert performance. Integrating functional MRI data could reveal how these structural changes translate into functional advantages during tasks. A multimodal imaging approach, combining structural, functional, and diffusion MRI, would provide a more comprehensive understanding of the neural mechanisms underlying motor expertise, allowing for a more nuanced exploration of how structural and functional changes interact to support high-level performance.

6. Conclusion

This study demonstrates that long-term golf training is associated with reorganized structural covariance networks, characterized by enhanced global modularity and local efficiency. Key sensorimotor and cognitive regions showed nodal-level changes, reflecting expertise-related neuroplasticity. Mediation analysis further revealed that local network properties, such as the clustering coefficient, bridge training duration and performance improvement. These findings suggest that prolonged motor training shapes brain architecture in ways that support superior skill execution and behavioral adaptation.

Reference

1. Dayan E, Cohen LG. Neuroplasticity subserving motor skill learning. Neuron. 2011;72(3):443-54. Epub 2011/11/15. doi: 10.1016/j.neuron.2011.10.008. PMID: 22078504.
2. Spampinato D, Celnik P. Multiple Motor Learning Processes in Humans: Defining Their Neurophysiological Bases. Neuroscientist. 2021;27(3):246-67. Epub 2020/07/28. doi: 10.1177/1073858420939552. PMID: 32713291.

3. Calmels C. Neural correlates of motor expertise: Extensive motor training and cortical changes. Brain Res. 2020;1739:146323. Epub 2019/07/07. doi: 10.1016/j.brainres.2019.146323. PMID: 31278937.
4. Paul DJ, Gabbett TJ, Nassis GP. Agility in Team Sports: Testing, Training and Factors Affecting Performance. Sports Med. 2016;46(3)(1179-2035 (Electronic)):421-42. doi: 10.1007/s40279-015-0428-2.
5. Wojtowicz M, Gardner AJ, Stanwell P, Zafonte R, Dickerson BC, Iverson GL. Cortical thickness and subcortical brain volumes in professional rugby league players. Neuroimage Clin. 2018;18:377-81. Epub 2018/03/01. doi: 10.1016/j.nicl.2018.01.005. PMID: 29487794.
6. Wei G, Luo J, Li Y. Brain structure in diving players on MR imaging studied with voxel-based morphometry. Progress in Natural Science. 2009;19(10):1397-402. doi: 10.1016/j.pnsc.2008.12.009.
7. Bezzola L, Mérillat S, Gaser C, Jäncke L. Training-Induced Neural Plasticity in Golf Novices. The Journal of Neuroscience. 2011;31(35):12444-8. doi: 10.1523/jneurosci.1996-11.2011.
8. Maguire EA, Woollett K, Spiers HJ. London taxi drivers and bus drivers: A structural MRI and neuropsychological analysis. Hippocampus. 2006;16(12):1091-101. doi: 10.1002/hipo.20233.
9. Li J, Cao Y, Huang M, Qin Z, Lang J. Progressive increase of brain gray matter volume in individuals with regular soccer training. Sci Rep. 2024;14(1):7023. Epub 2024/03/26. doi: 10.1038/s41598-024-57501-4. PMID: 38528027.
10. Park IS, Lee Kj Fau - Han JW, Han Jw Fau - Lee NJ, Lee Nj Fau - Lee WT, Lee Wt Fau - Park KA, Park Ka Fau - Rhyu IJ, et al. Basketball training increases striatum volume. Hum Mov Sci. 2011 30(1)(1872-7646 (Electronic)):56-62. doi: 10.1016/j.humov.2010.09.001.
11. Park IS, Lee Kj Fau - Han JW, Han Jw Fau - Lee NJ, Lee Nj Fau - Lee WT, Lee Wt Fau - Park KA, Park Ka Fau - Rhyu IJ, et al. Experience-dependent plasticity of cerebellar vermis in basketball players. Cerebellum. 2009 8(3)(1473-4230 (Electronic)):334-9. doi: 10.1007/s12311-009-0100-1.
12. Teixeira-Machado L, Arida RM, de Jesus Mari J. Dance for neuroplasticity: A descriptive systematic review. Neuroscience & Biobehavioral Reviews. 2019;96:232-40. doi: https://doi.org/10.1016/j.neubiorev.2018.12.010.
13. Nigmatullina Y, Hellyer PJ, Nachev P, Sharp DJ, Seemungal BM. The neuroanatomical correlates of training-related perceptuo-reflex uncoupling in dancers. Cereb Cortex. 2013 25(2):554–62. doi: 10.1093/cercor/bht266.
14. Huang R, Lu M, Song Z, Wang J. Long-term intensive training induced brain structural changes in world class gymnasts. Brain Structure and Function. 2015;220(2):625-44. doi: 10.1007/s00429-013-0677-5.
15. Fukuo M, Kamagata K, Kuramochi M, Andica C, Tomita H, Waki H, et al. Regional brain gray matter volume in world-class artistic gymnasts. The Journal of Physiological Sciences. 2020;70(1):43. doi: https://doi.org/10.1186/s12576-020-00767-w.
16. Rubinov M, Sporns O. Complex network measures of brain connectivity: uses and interpretations. Neuroimage. 2010;52(3):1059-69. Epub 2009/10/13. doi: 10.1016/j.neuroimage.2009.10.003. PMID: 19819337.
17. Wang J, Lu M, Fan Y, Wen X, Zhang R, Wang B, et al. Exploring brain functional plasticity in world class gymnasts: a network analysis. Brain Struct Funct. 2016;221(7):3503-19. Epub 2015/10/01. doi: 10.1007/s00429-015-1116-6. PMID: 26420277.
18. Shao X, Luo D, Zhou Y, Xiao Z, Wu J, Tan LH, et al. Myeloarchitectonic plasticity in elite golf players' brains. Hum Brain Mapp. 2022;43(11):3461-8. Epub 2022/04/15. doi: 10.1002/hbm.25860.

PMID: 35420729.
19. Huang SY, Hsu JL, Lin KJ, Hsiao IT. A Novel Individual Metabolic Brain Network for 18F-FDG PET Imaging. Front Neurosci. 2020;14:344. Epub 2020/06/02. doi: 10.3389/fnins.2020.00344. PMID: 32477042.
20. Ping L, Sun S, Zhou C, Que J, You Z, Xu X, et al. Altered topology of individual brain structural covariance networks in major depressive disorder. Psychol Med. 2023:1-12. Epub 2023/07/10. doi: 10.1017/S003329172300168X. PMID: 37427670.
21. Sporns O, Zwi JD, Sporns O, Zwi JD. The Small World of the Cerebral Cortex. Neuroinformatics. 2004;2(2):145-62. doi: 10.1385/NI:2:2:145.
22. Cao L, Li L, Huang Z, Xia F, Huang R, Ma Y, et al. Functional network segregation is associated with higher functional connectivity in endurance runners. Neuroscience Letters. 2023;812(24):137401. doi: 10.1016/j.neulet.2023.137401.
23. Li J, Cao Y, Huang M, Li Z, Qin Z, Lang J. The alterations of functional brain networks and its relationship with sport decision-making and training duration in soccer players across different skill levels. Neuroscience Letters. 2024;831:137788. doi: https://doi.org/10.1016/j.neulet.2024.137788.
24. Wenger E, Brozzoli C, Lindenberger U, Lövdén M. Expansion and Renormalization of Human Brain Structure During Skill Acquisition. Trends in Cognitive Sciences. 2017;21(12):930-9. doi: https://doi.org/10.1016/j.tics.2017.09.008.
25. Calmels C. Neural correlates of motor expertise: Extensive motor training and cortical changes. Brain Research. 2020;1739:146323. doi: https://doi.org/10.1016/j.brainres.2019.146323.
26. Draganski B, Gaser C, May A, Draganski B, Gaser C, Busch V, et al. Changes in grey matter induced by training. Nature. 2004;427(6972):311-2. doi: 10.1038/427311a.
27. Li J, Cao Y, Huang M, Qin Z, Lang J. Progressive increase of brain gray matter volume in individuals with regular soccer training. Scientific Reports. 2024;14(1):7023. doi: 10.1038/s41598-024-57501-4.
28. Vázquez-Rodríguez BA-O, Suárez LE, Markello RD, Shafiei G, Paquola C, Hagmann P, et al. Gradients of structure-function tethering across neocortex. Proc Natl Acad Sci U S A. 2019;116(42):21219-27.
29. Popp JL, Thiele JA, Faskowitz J, Seguin C, Sporns O, Hilger K. Structural-functional brain network coupling predicts human cognitive ability. Neuroimage. 2024;290:120563.
30. Suárez LE, Markello RD, Betzel RF, Misic B. Linking Structure and Function in Macroscale Brain Networks. Trends Cogn Sci. 2020;24(4):302-15.
31. Yao Z, Hu B, Zheng J. A review of structural and functional brain networks: small world and atlas. Brain informatics. 2015;2(1):45-52.
32. Park H-J, Friston K. Structural and Functional Brain Networks: From Connections to Cognition. Science. 2013;342(6158):1238411. doi: doi:10.1126/science.1238411.
33. Li LX, Smith DM, Li L, Smith DM. Neural Efficiency in Athletes: A Systematic Review. Frontiers in behavioral neuroscience. 2021;15(12):698555. doi: 10.3389/fnbeh.2021.698555.
34. Hüfner K, Binetti C, Hamilton DA, Stephan T, Flanagin VL, Linn J, et al. Structural and functional plasticity of the hippocampal formation in professional dancers and slackliners. Hippocampus. 2011;21(8):855-65. PMID: 898675503; 2011-18279-006.
35. Wei GX, Zhang YC, Luo J, Wei G, Zhang Y, Jiang T, et al. Increased Cortical Thickness in Sports Experts: A Comparison of Diving Players with the Controls. PloS one. 2011;6(2):e17112. doi:

10.1371/journal.pone.0017112.
36. Pascual B, Masdeu JC, Dickerson BC, Pascual B, Masdeu JC, Hollenbeck M, et al. Large-Scale Brain Networks of the Human Left Temporal Pole: A Functional Connectivity MRI Study. Cerebral cortex. 2015;25(3):680-702. doi: 10.1093/cercor/bht260.
37. Hüfner K, Strupp M, Jahn K, Hüfner K, Strupp M, Smith P, et al. Spatial separation of visual and vestibular processing in the human hippocampal formation. Annals of the New York Academy of Sciences. 2011;1233(1):177-86. doi: 10.1111/j.1749-6632.2011.06115.x.
38. Glosser G, Salvucci AE, Chiaravalloti ND, Glosser G, Salvucci AE, Chiaravalloti ND. Naming and recognizing famous faces in temporal lobe epilepsy. Neurology. 2003;61(1):81-6. doi: 10.1212/01.WNL.0000073621.18013.E1.
39. Olson IR, Ploaker A, Ezzyat Y, Olson IR, Plotzker A, Ezzyat Y. The Enigmatic temporal pole: a review of findings on social and emotional processing. Brain. 2007;130(7):1718-31. doi: 10.1093/brain/awm052.
