# Supplementary material for: Long-term intensive golf training induces reconfiguration of brain structural covariance networks
Source: PLoS One. 2026 Mar 9;21(3):e0344165. doi: 10.1371/journal.pone.0344165 (PMC12970903; doi:10.1371/journal.pone.0344165)
Supplement: Attachment — Submitted filename: Response to Reviewers.docx [file pone.0344165.s002.docx]

Response to Reviewers

Editor and Reviewers,

Thank you for the opportunity to revise our manuscript titled “Long-Term Intensive Golf Training Induces Reconfiguration of Brain Structural Covariance Networks” (PONE-D-25-36556R1). We sincerely appreciate the reviewers’ insightful and constructive comments, which have significantly helped us improve the quality and clarity of the manuscript.

We have carefully addressed all the points raised in the review, particularly those by Reviewer #1, and have made corresponding revisions throughout the text. In the revised manuscript, all changes are red color.

Below, we detail our point-by-point responses to the reviewers’ comments.

**Reviewer #1:**

**1. Clarify statistical assumptions. Briefly specify whether the data met the assumptions for parametric testing and how correlation types were chosen.**

**Response**: We thank the reviewer for this suggestion. We have now clarified the statistical assumptions in the Statistical Analysis section (Section 2.5). Specifically, we have added that: The permutation tests used for group comparisons are non-parametric and do not assume normality, providing a robust and distribution-free assessment. Prior to correlation analyses, we assessed normality using the Shapiro–Wilk test. Variables conforming to normality were analyzed with Pearson’s correlation, otherwise Spearman’s correlation was used. In this study, all variables met normality assumptions, so Pearson’s correlation was applied.

2. **Data availability. Consider depositing anonymized SCN matrices or summary statistics in a public repository, even under restricted access.**

**Response**:We thank the reviewer for this valuable suggestion. In response to your comment, we have now publicly deposited the anonymized individual structural covariance network (SCN) matrices and all derived network metrics, along with the complete analysis code, in a GitHub repository. The repository is publicly accessible without restriction. We have also included a Data Availability Statement at the end of the manuscript that provides the direct link to this repository, ensuring full transparency and compliance with PLOS ONE's data policy.

3. **Minor linguistic refinements. The Discussion section could be slightly condensed to avoid repetition and improve flow.**

**Response**: We thank the reviewer for the suggestion. We have carefully revised the Discussion section to improve clarity, reduce redundancy, and enhance logical flow

4. **Terminology consistency. Ensure uniform usage of abbreviations (e.g., Cp, Eloc, Eg) throughout figures and text.**

**Response**: We have thoroughly checked the entire manuscript, figures, and tables to ensure consistent use of abbreviations for network metrics

**Reviewer #2:**

This is a well-executed and articulate study that has made novel contributions to knowledge on experience-dependent reorganization of the brain network associated with motor skills mastery. I have found the manuscript free of remaining concerns and can be published as it is.

Response: We sincerely thank the reviewer for the positive evaluation and endorsement of our work. We are pleased that the reviewer found the manuscript articulate and novel. In response to Reviewer #1’s comments, we have made several refinements to further improve clarity and rigor, which we believe strengthen the manuscript without altering its core contributions.
