# Supplementary material for: Long-term intensive golf training induces reconfiguration of brain structural covariance networks
Source: PLoS One. 2026 Mar 9;21(3):e0344165. doi: 10.1371/journal.pone.0344165 (PMC12970903; doi:10.1371/journal.pone.0344165)
Supplement: Attachment — Submitted filename: Response_to_Reviewers_auresp_3.docx [file pone.0344165.s003.docx]

**Reviewer #1:**

2. Participant Number Discrepancy:In the Abstract (according to the file snippet/metadata), it states: "A total of 60 participants—30 expert golfers, 30 novice golfers, and 30 non-golfer controls." (Note: 30+30+30=90, not 60).In Section 2.1 (Subjects), it states: "The Pro group comprised 20... The New group included 20... The control group consisted of 20." (Total 60). Ensure the numbers in the Abstract match the Methods exactly (likely 20 per group).

**Re:** Thank you for catching this discrepancy. The correct sample size is 20 per group, totaling 60 participants. We have revised the Abstract accordingly to state “20 expert golfers, 20 novice golfers, and 20 non-golfer controls” and removed the inconsistent numbers. The Methods section remains unchanged.

3. Contradiction in Mediation Analysis (Partial vs. Full). You state the clustering coefficient "partially mediated the association."Results (Section 3.3): You state: "The direct effect of training duration on stroke performance became non-significant after controlling for Cp. Check your statistical output. If the direct path is indeed non-significant (p > .05), change the Abstract to say "fully mediated" or simply "mediated". If it is significant but reduced, change the Results text to reflect that.

**Re:** Thank you for your careful reading and for pointing out the imprecise wording regarding the mediation result. We have revised the abstract accordingly.

In the Results section (3.3), we reported that the direct effect of training duration on stroke performance became non-significant (β = −0.22, p = 0.15) after including the clustering coefficient (Cp) as a mediator, while the indirect effect was significant (β = −0.1, 95% CI [−0.37, −0.09]). According to standard terminology, this pattern indicates full mediation, not partial mediation.

We have now corrected the abstract to state that the clustering coefficient “mediated” the association, removing the word “partially” to accurately reflect the statistical finding. The revised sentence now reads:

“Importantly, the clustering coefficient mediated the association between training duration and stroke accuracy.”

4. Anatomical Terminology Error ("Crus 10"): You refer to "right cerebellar Crus 10" (or Crus X). Results (3.2): You refer to "right cerebellum-10". Change "Crus 10" in the Discussion to "Lobule X" or "Cerebellum Region 10" to be anatomically correct.

**Re：**Thank you for pointing out the anatomical error. We have corrected “right cerebellum-10” and “Crus 10” to “right Cerebellum Region 10” throughout the manuscript, tables, and figures to align with AAL atlas conventions.

5. Ensure you explicitly argue why reduced integration in the visual cortex helps. (e.g., "The reduction in global efficiency may reflect a pruning of irrelevant connections to prevent visual distractors, focusing resources on local processing...")

**Re：**Thank you for your comment. We agree that merely reporting reduced global efficiency in the left calcarine cortex is insufficient; we need to explicitly argue why this reduction is functionally beneficial.

We have revised the Discussion accordingly. The new paragraph interprets this decrease as a functional pruning process, whereby long-term training suppresses task-irrelevant visual connections to enhance focal attention and reduce perceptual noise—critical demands in golf. We now also cite evidence from gymnasts and shooters showing similar visual cortex reductions alongside superior performance, supporting this as an adaptive neural efficiency effect rather than degradation. The revised text is included in Section 4.
